# Is there a difference in catheter-related thrombosis between left- and right-sided arm ports and chest ports?

**DOI:** 10.3389/fsurg.2026.1735554

**Published:** 2026-02-20

**Authors:** Yun Fan, Huarong Du, Yuanyuan Guan, Aili Zhang, Xiaolin Jiang

**Affiliations:** 1 Department of Gastrointestinal and Rectal Surgery, Shapingba Hospital Affiliated to Chongqing University (Shapingba District People’s Hospital of Chongqing), Chongqing, China; 2 Department of Quality Management, Shapingba Hospital Affiliated to Chongqing University (Shapingba District People’s Hospital of Chongqing), Chongqing, China; 3 Department of Oncology, Army Medical Center of PLA, Chongqing, China; 4 Department of Oncology, Songshan General Hospital, Chongqing, China

**Keywords:** arm port, catheter-related thrombosis, chest port, meta-analysis, totally implantable venous access ports, venous thrombosis

## Abstract

**Background:**

Totally Implantable Venous Access Ports (TIVAPs) are long-term subcutaneous venous infusion devices widely used in patients requiring prolonged venous therapy, particularly those with cancer. The choice of left- vs. right-sided implantation during TIVAP implantation is a key clinical decision, as anatomical and hemodynamic differences between sides may influence the risk of catheter-related thrombosis (CRT). However, existing literature remains controversial regarding the association between implantation side and CRT incidence. This meta-analysis aims to systematically evaluate the impact of left- vs. right-sided TIVAP implantation on CRT risk, providing evidence-based support for clinical prevention strategies.

**Methods:**

Literature searches were conducted in PubMed, Web of Science, Embase, Cochrane Library, Chinese Biomedical Literature Database (CBM), China National Knowledge Infrastructure (CNKI), Wanfang Data, and VIP Database to identify studies investigating the effect of left- vs. right-sided TIVAP implantation on CRT incidence. The search spanned from database inception to October 2025. Two independent researchers screened literature, extracted data, and assessed the risk of bias of the included studies. A meta-analysis was conducted using RevMan 5.3 software.

**Results:**

A total of 21 studies involving 10,778 patients were included. Meta-analysis revealed no statistically significant difference in CRT incidence between left- and right-sided chest ports [OR = 1.28, 95%CI (0.97–1.68), *P* = 0.08] or arm port [OR = 1.19,95% CI (0.86–1.66), *P* = 0.29].

**Conclusions:**

Current evidence indicates no overall difference in CRT incidence between left- and right-sided TIVAPs. However, the observed sample size-dependent association suggests that left-sided implantation may carry a slightly higher CRT risk in large cohorts. Clinicians may select the implantation side based on individual patient characteristics. However, large-sample, multi-center randomized controlled trials are needed to further validate these findings, particularly given the observed sample size-dependent differences.

## 1 Introduction

Totally Implantable Venous Access Ports (TIVAPs) are subcutaneous, long-term venous infusion devices that provide safe, convenient, and durable vascular access for patients requiring prolonged therapy—most commonly individuals with cancer (1). Composed of a puncturable injection port and a venous catheter, TIVAPs are classified as chest ports or arm ports based on the puncture site (2, 3). Compared to traditional peripheral venous catheters, TIVAPs reduce complications such as repeated puncture pain, drug extravasation, and phlebitis, while enabling patients to maintain mobility during treatment intervals, thereby improving patients’ quality of life and treatment adherence (4–6).

Despite these advantages, catheter-related thrombosis (CRT) remains a major concern with TIVAP use (7, 8). CRT causes local symptoms such as swelling and pain, impairs treatment continuity, and may lead to life-threatening complications including pulmonary embolism (9, 10). Cancer patients are particularly vulnerable to CRT due to disease-related hypercoagulability, surgical trauma, and prolonged immobility (11). Research reports indicated that the incidence of CRT caused by infusion ports ranges from 1.04% to 15.8% (12), which may result in device removal, increased healthcare costs, and prolonged treatment duration. In severe cases, it may even directly endanger the patient's life. Thus, identifying strategies to prevent TIVAP-related CRT is a critical focus of clinical research.

Anatomical and hemodynamic differences between the left and right sides of the body have led to speculation that TIVAP implantation side may influence CRT risk (13–15). The right internal jugular vein is larger and forms a nearly straight line with the brachiocephalic vein and superior vena cava, facilitating unobstructed blood flow. In contrast, the acute angle between the left brachiocephalic vein and superior vena cava may slow blood flow and create vortices, and promote platelet aggregation and thrombosis. Some studies have reported a higher CRT risk with left-sided TIVAP implantation, while others have found no significant association between implantation side and CRT incidence (16–19). This inconsistency has created uncertainty for clinicians selecting TIVAP implantation sides, highlighting the need for evidence-based guidelines.

Clarifying the relationship between TIVAP implantation side and CRT risk would provide valuable guidance for clinical practice, helping minimize thrombosis risk and optimize patient outcomes. Therefore, this meta-analysis systematically synthesized existing evidence to evaluate the impact of left- vs. right-sided TIVAP implantation (chest and arm ports) on CRT incidence.

## 2 Methods

### 2.1 Search strategy

We retrieved literature on the impact of left and right sides implantation of infusion ports on the incidence of catheter-related thrombosis by searching PubMed, Web of Science, Embase, Cochrane Library, Chinese Biomedical Literature Database (CBM), China National Knowledge Infrastructure (CNKI), Wanfang, and VIP databases. The search period was from the establishment of the databases to October 2025. A combination of MeSH terms and free-text keywords were used to maximize search sensitivity and specificity (Supplementary Table S1). The search strategy included terms related to TIVAPs (e.g., “Port,” “TIVAP,” “Vascular Access Port”) and CRT (e.g., “Venous thrombosis,” “Catheter-related thrombosis,” “Thrombus”).

### 2.2 Inclusion and exclusion criteria

#### 2.2.1 Inclusion criteria

1. Study Design: Case-control studies, cohort studies, or randomized controlled trials (RCTs);
2. Participants: Patients who underwent chest port or arm port implantation;
3. Exposure factor: Comparison of left-sided vs. right-sided TIVAP implantation;
4. Outcomes: Incidence of CRT.

#### 2.2.2 Exclusion criteria

1. Duplicate publications;
2. Studies without full-text availability;
3. Studies with insufficient data for extraction;
4. Review, case series, conference paper, animal experiment, or non-original research.

### 2.3 Data extraction

Literature was imported into EndNote X9 software for duplicate removal. Two independent researchers first screened titles and abstracts to exclude irrelevant studies, and then conducted full-text reviews against the inclusion/exclusion criteria. Disagreements were resolved by consensus with a third senior researcher. Extracted data included: first author, publication year, country, study design, sample size, TIVAP type (chest/arm port), number of left- and right-sided implantations, patient age and gender, data collection period, and CRT incidence.

### 2.4 Risk of bias assessment

Two independent researchers assessed the risk of bias across all included studies using the Risk Of Bias In Non-randomized Studies of Interventions (ROBINS-I) tool. Any discrepancies in their evaluations were resolved through adjudication by a third senior researcher. This validated assessment tool comprises 7 bias domains and 33 signaling questions, each of which was answered in a standardized sequential format with the following categorical responses: Yes (Y), Probably yes (PY), No (N), Probably no (PN), and No information (NI). Risk of bias judgments for each study were derived from the systematic responses to these signaling questions and stratified into five hierarchical levels: Low risk, Moderate risk, High risk, Very high risk, and No information.

### 2.5 Data synthesis and analysis

Statistical analysis was performed using RevMan 5.3 software. The odds ratio (OR) and its 95% confidence interval (CI) were used as the effect size to compare CRT incidence between left- and right-sided TIVAPs. Heterogeneity was assessed using the *I*^2^ test and chi-square test. If *I*^2^ < 50% and *P* *>* *0.1*, a fixed-effect model was used. If *I*^2^ ≥ 50% and *P* ≤ 0.1, a random-effect model was used (20, 21). Subgroup analyses were conducted by country (China vs. other countries) and sample size (≤500 vs. >500). Sensitivity analysis was performed by sequentially excluding each study to evaluate the robustness of results. Publication bias was assessed using funnel plots.

## 3 Results

### 3.1 Study characteristics

**Figure 1:**
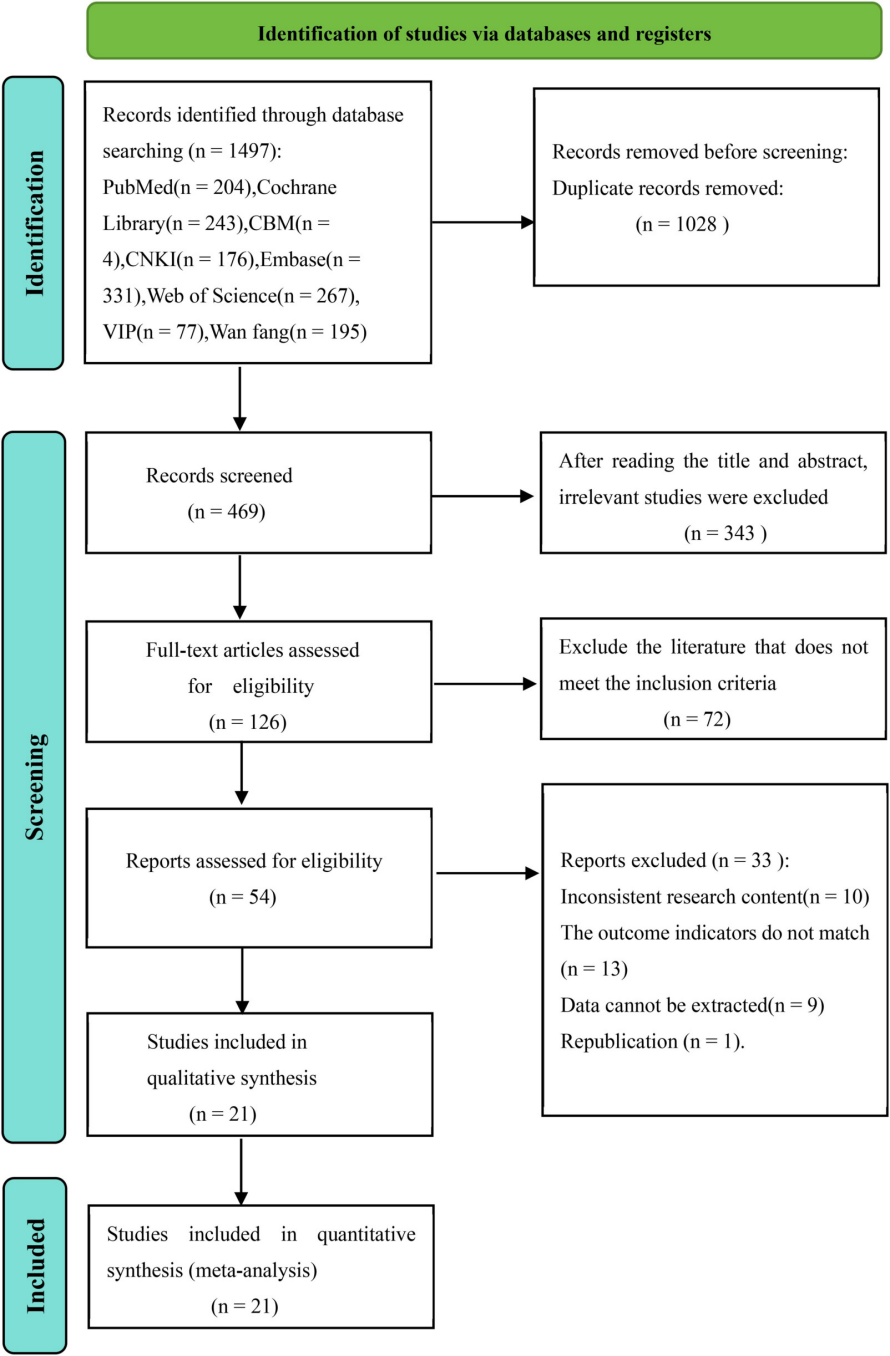
Flowchart onnnf database search and study inclusion.

The literature search yielded 1,497 records, of which 1,028 duplicates were removed. After screening titles/abstracts and full texts, 21 studies (13–15, 17–19, 22–36) were included in the meta-analysis (Figure 1). These studies were published between 2007 and 2024, involving 10,778 patients (3391 with left-sided TIVAPs and 7387 with right-sided TIVAPs). Sample sizes ranged from 72 to 2,104. Thirteen studies focused on chest ports, and eight on arm ports. Most studies were conducted in China (18 studies), with three from other countries (Germany, Canada, and the United States). Detailed study characteristics are summarized in Table 1. Despite including cohort studies and RCTs in our initial eligibility criteria, no such studies met all inclusion requirements (e.g., sufficient data on implantation side and CRT incidence), leading to the inclusion of only case-control studies. All included studies were case-control designs. The ROBINS-I assessment revealed 8 studies with low bias risk, 11 with moderate bias risk, and 2 with high bias risk (Table 2).

**Table 1:** The basic characteristics of the included literature.

Study	Country	Study design	Sample size	The number of patients on the left	The number of patients on the right	Type of infusion port	Gender(male/Female)	Age (years)	Date collection time
Chen et al. 2017	China	Case-control	190	35	155	Chest port	0/190	46.62 ± 8.33	April 2014 to April 2015
Guan et al. 2018	China	Case-control	101	21	80	Chest port	0/101	48.3 ± 7.1	January 2017 to December 2017
Han et al. 2023	China	Case-control	960	390	570	Chest port	0/960	24-71	January 2017 to October 2019
Liu et al. 2017	China	Case-control	755	281	474	Chest port	0/755	48.17	January 2014 to March 2016
Lou et al. 2020	China	Case-control	541	300	241	Chest port	0/541	48.75 ± 8.64	January 2017 to December 2019
Shi et al. 2015	China	Case-control	172	48	124	Chest port	0/172	21-76	November 2013 to December 2014
Xu et al. 2020	China	Case-control	770	369	401	Arm port	0/770	–	May 2016 to September 2019
Zhang et al. 2022	China	Case-control	183	30	153	Chest port	–	–	March 2019 to June 2021
Guo et al. 2020	China	Case-control	510	93	417	Arm port	287/223	60.85 ± 5.26	January 1, 2017 to December 1, 2019
Qin et al. 2024	China	Case-control	2104	201	1903	Chest port	412/1692	50.4 ± 15.6	May 2019 to December 2022
Yu et al. 2024	China	Case-control	419	11	408	Chest port	–	–	February 2013 to November 2018
Tsuruta et al. 2019	Germany	Case-control	482	57	425	Chest port	251/231	–	April 2012 to December 2017
Suleman et al. 2019	Canada	Case-control	389	63	326	Arm port	123/266	58.2	August 2015 to September 2017
Hong et al. 2019	US	Case-control	176	65	111	Arm port	136/38	19-84	May 2012 to June 2015
Ren 2022	China	Case-control	331	187	144	Arm port	–	52.66 ± 8.16	January 2018 to November 2021
Liao et al. 2020	China	Case-control	1595	676	919	Chest port	0/1595	50. 4 ± 9. 4	From 2017 to 2019
Chen 2019	China	Case-control	312	172	140	Chest port	0/312	50.52 ± 8.72	January 2016 to September 2016
Huang et al. 2024	China	Case-control	120	65	55	Arm port	–	–	February 2021 to February 2022
Hu et al. 2023	China	Case-control	72	7	65	Arm port	44/28	–	June 2020 to June 2022
Goltz et al. 2012	Germany	Case-control	200	155	45	Arm port	94/106	57.7 ± 14	March 2010 to March 2013
Makary et al. 2018	US	Case-control	396	165	231	Chest port	0/396	53.1 ± 10.8.	January 2007 to December 2013

**Table 2:** Summary of bias assessment using the ROBINS-I tool.

Study	D1	D2	D3	D4	D5	D6	D7	Overall bias
Chen et al. 2017	Low	Low	Low	Low	Low	Low	Low	Low
Guan et al. 2018	Low	Low	Low	Low	Low	Moderate	Low	Moderate
Han et al. 2023	Low	Low	Low	Low	Low	Low	Moderate	Moderate
Liu et al. 2017	Low	Low	Low	Low	Low	Low	Low	Low
Lou et al. 2020	Low	Moderate	Low	Low	Moderate	High	Low	High
Shi et al. 2016	Moderate	Low	Low	Low	Low	Low	Low	Moderate
Xu et al. 2020	Moderate	Low	Low	Low	High	Low	Low	High
Zhang et al. 2022	Moderate	Low	Low	Low	Low	Low	Low	Moderate
Guo et al. 2020	Low	Low	Low	Low	Low	Low	Low	Low
Qin et al. 2024	Low	Low	Moderate	Low	Low	Moderate	Low	Moderate
Yu et al. 2024	Low	Low	Low	Low	Low	Low	Low	Low
Tsuruta et al. 2019	Low	Moderate	Low	Low	Low	Low	Low	Moderate
Suleman et al. 2019	Low	Moderate	Low	Low	Low	Low	Low	Moderate
Hong et al. 2019	Moderate	Low	Low	Low	Low	Low	Moderate	Moderate
Ren 2022	Low	Low	Low	Low	Low	Low	Low	Low
Liao et al. 2020	Low	Low	Moderate	Low	Low	Low	Low	Moderate
Chen 2019	Low	Low	Low	Low	Low	Low	Low	Low
Huang et al. 2024	Low	Low	Low	Low	Low	Low	Low	Low
Hu et al. 2023	Low	Low	Low	Low	Moderate	Low	Low	Moderate
Goltz et al. 2012	Low	Moderate	Low	Low	Low	Low	Low	Moderate
Makary et al. 2018	Low	Low	Low	Low	Low	Low	Low	Low

Domains:

D1:Bias due to confounding.

D2:Bias in selection of participants.

D3:Bias in classification of exposures.

D4:Bias due to deviations from intended exposures.

D5:Bias due to missing data.

D6:Bias in measurement of outcomes.

D7:bias in selection of the reported result.

### 3.2 Meta-analysis

#### 3.2.1 Chest ports: left vs., right CRT incidence

**Figure 2:**
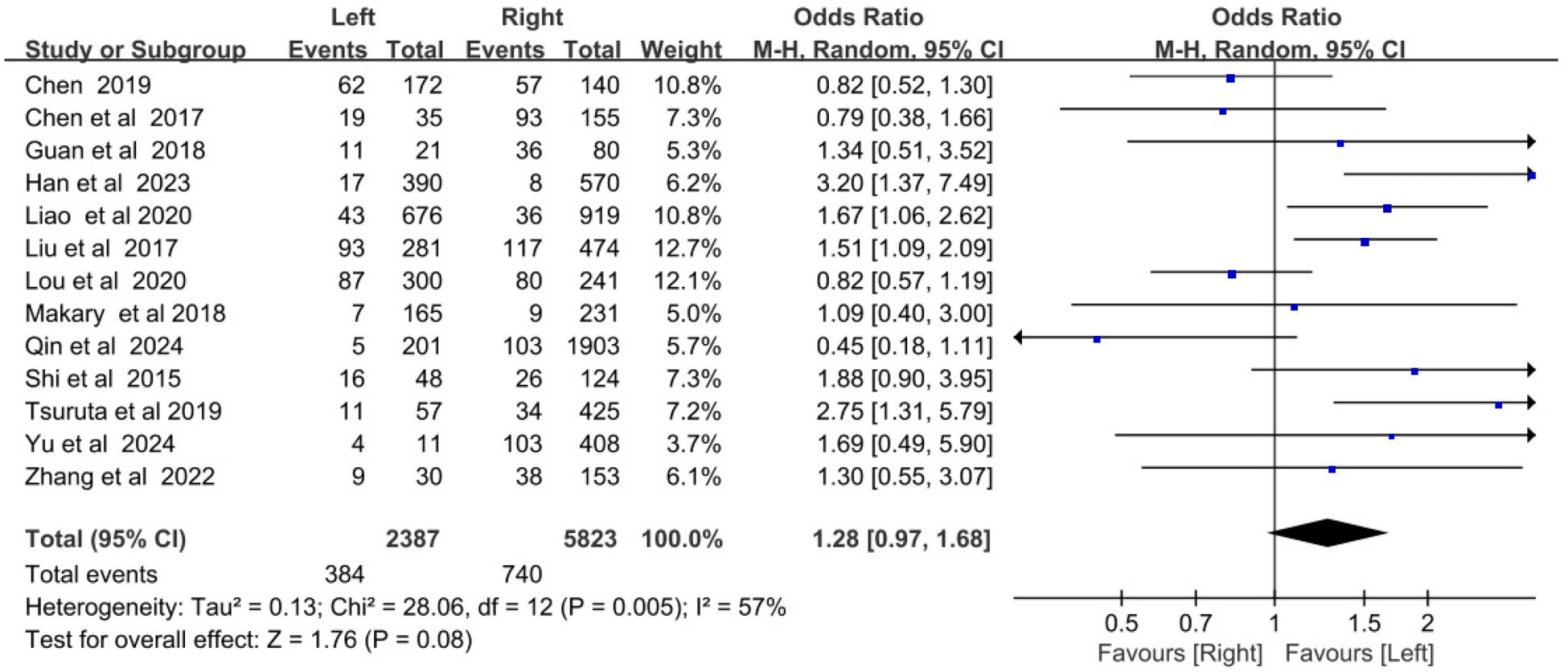
Forest plot comparing the incidence of catheter-related thrombosis on the left and right sides of the chest port.

Thirteen studies reported CRT incidence in left- and right-sided chest ports. Heterogeneity was moderate (*I*^2^ = 57%, *P* *=* *0.08*), so a random-effects model was adopted. Meta-analysis showed no statistically significant difference in CRT incidence between left- and right-sided chest ports [OR = 1.28, 95% CI (0.97–1.68), *P* = 0.08] (Figure 2).

#### 3.2.3 Arm ports: left vs., right CRT incidence

**Figure 3:**
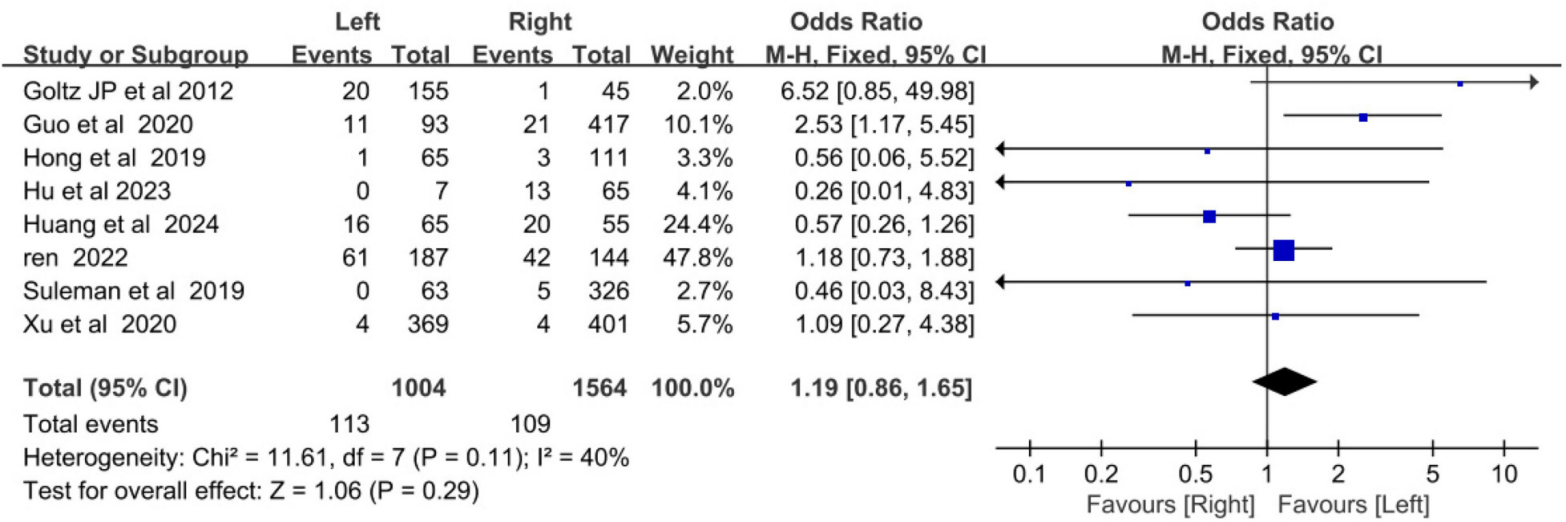
Forest plot comparing the incidence of catheter-related thrombosis on the left and right sides of the arm port.

Eight studies reported CRT incidence in left- and right-sided arm ports. Heterogeneity was low (*I*^2^ = 40%, *P* *=* *0.29*), so a fixed-effects model was used. No statistically significant difference in CRT incidence was observed between left- and right-sided arm ports [OR = 1.19, 95%CI (0.86–1.66), *P* = 0.29] (Figure 3).

### 3.3 Subgroup analysis

We conducted subgroup analyses based on country (China vs. Others) and sample size (≤500 vs. >500) (Table 3).

**Table 3:** The pooled results of each outcomes.

Subgroup analysis	No. of studies	Heterogeneity		OR(95%CI)	*P*-value
		*I* ^2^	*P*		
Chest port					
Sample size					
≤500	8	33%	0.16	1.12 (0.90-1.54)	0.23
>500	5	77%	0.002	**1.23 (1.01–1.50)**	**0.04**
Country					
China	11	57%	0.009	1.21 (0.91–1.61)	0.19
Others	2	53%	0.15	1.85 (0.75–4.56)	0.18
Arm port					
Sample size					
≤500	6	29%	0.22	1.04 (0.72–1.50)	0.84
>500	2	8%	0.3	**2.01 (1.02–3.98)**	**0.04**
Country					
China	5	51%	0.09	1.12 (0.62–2.01)	0.71
Others	3	43%	0.18	1.40 (0.23–8.54)	0.72

Bold values indicate that the difference was statistically significant.

#### 3.3.1 Stratification by country

China: For arm ports, the pooled OR was 1.12 (95% CI: 0.62–2.01, *P* *=* *0.71*); for chest ports, the pooled OR was 1.21 (95% CI: 0.91–1.61, *P* *=* *0.19*). No significant differences were observed.

Other countries: For arm ports, the pooled OR was 1.40 (95% CI: 0.23–8.54, *P* *=* *0.72*); for chest ports, the pooled OR was 1.85 (95% CI: 0.75–4.56, *P* *=* *0.18*). No significant differences were observed.

#### 3.3.2 Stratification by sample size

Sample size >500: Left-sided arm ports had a significantly higher CRT incidence than right-sided ports (OR = 2.01, 95% CI: 1.02–3.98, *P* *=* *0.04*). Similarly, left-sided chest ports had a significantly higher CRT incidence (OR = 1.23, 95% CI: 1.01–1.50, *P* *=* *0.04*)

Sample size ≤500: For arm ports, the pooled OR was 1.04 (95% CI: 0.72–1.50, *P* *=* *0.84*); for chest ports, the pooled OR was 1.12 (95% CI: 0.90–1.54, *P* *=* *0.23*). No significant differences were observed.

### 3.4 Sensitivity analysis

**Figure 4:**
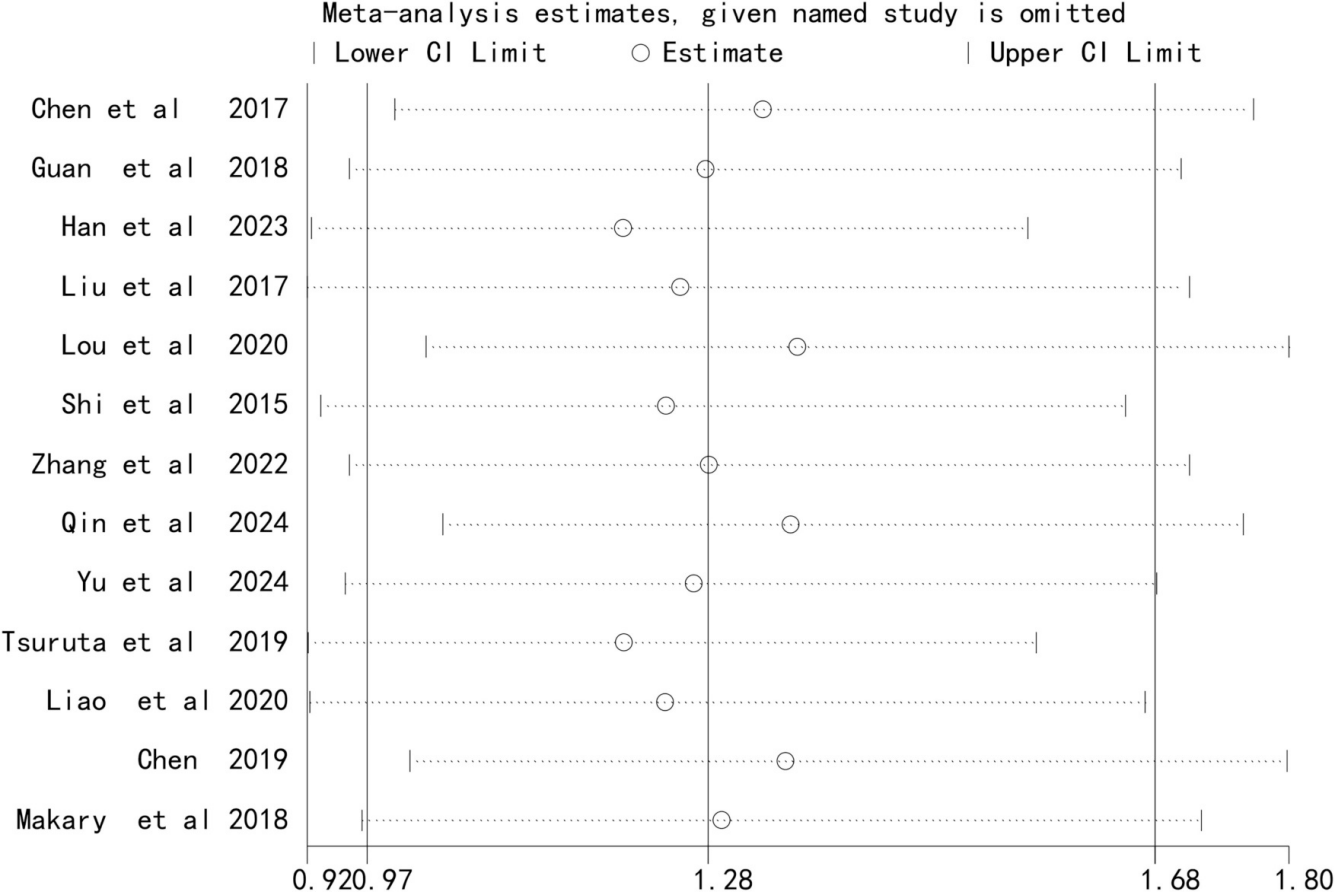
Sensitivity analysis comparing the incidence of catheter-related thrombosis on the left and right sides of the chest port.

**Figure 5:**
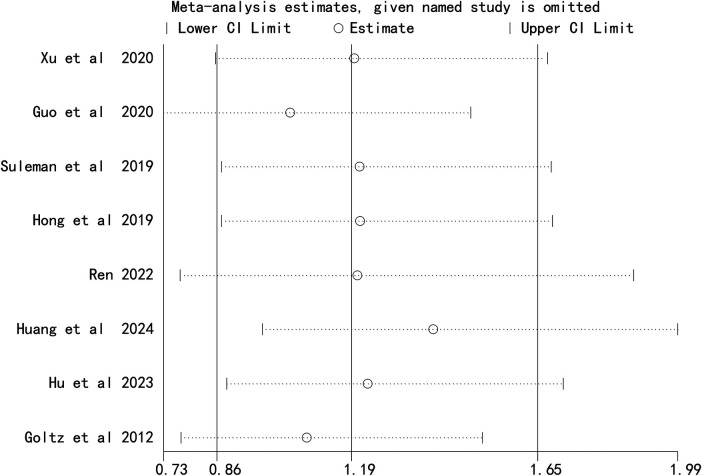
Sensitivity analysis comparing the incidence of catheter-related thrombosis on the left and right sides of the arm port.

Sequential exclusion of each study did not substantially alter the combined effect sizes for chest ports (Figure 4) or arm ports (Figure 5), indicating the robustness of the meta-analysis results.

### 3.5 Publication bias

**Figure 6:**
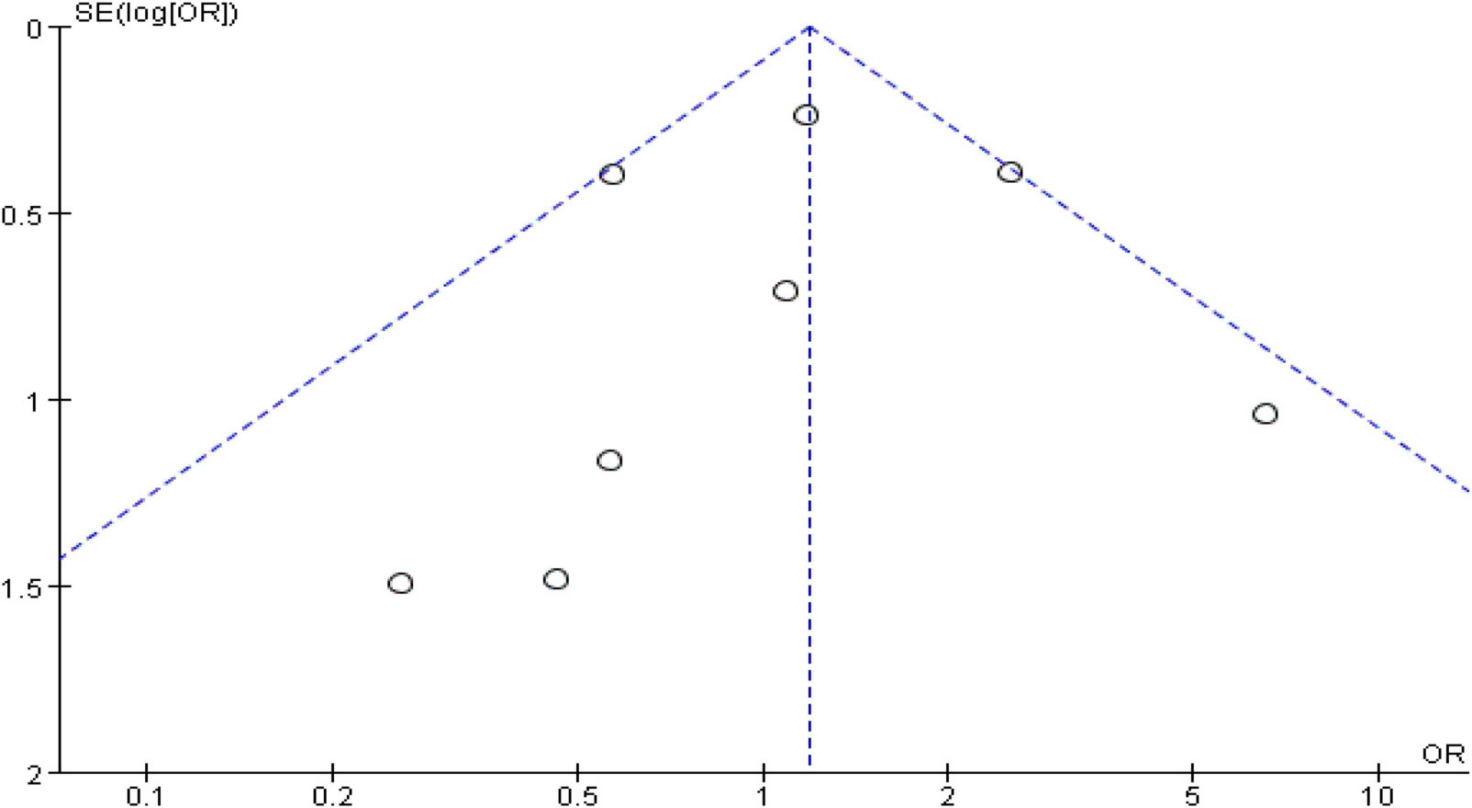
Funnel plots comparing the incidence of catheter-related thrombosis on the left and right sides of the chest port.

**Figure 7:**
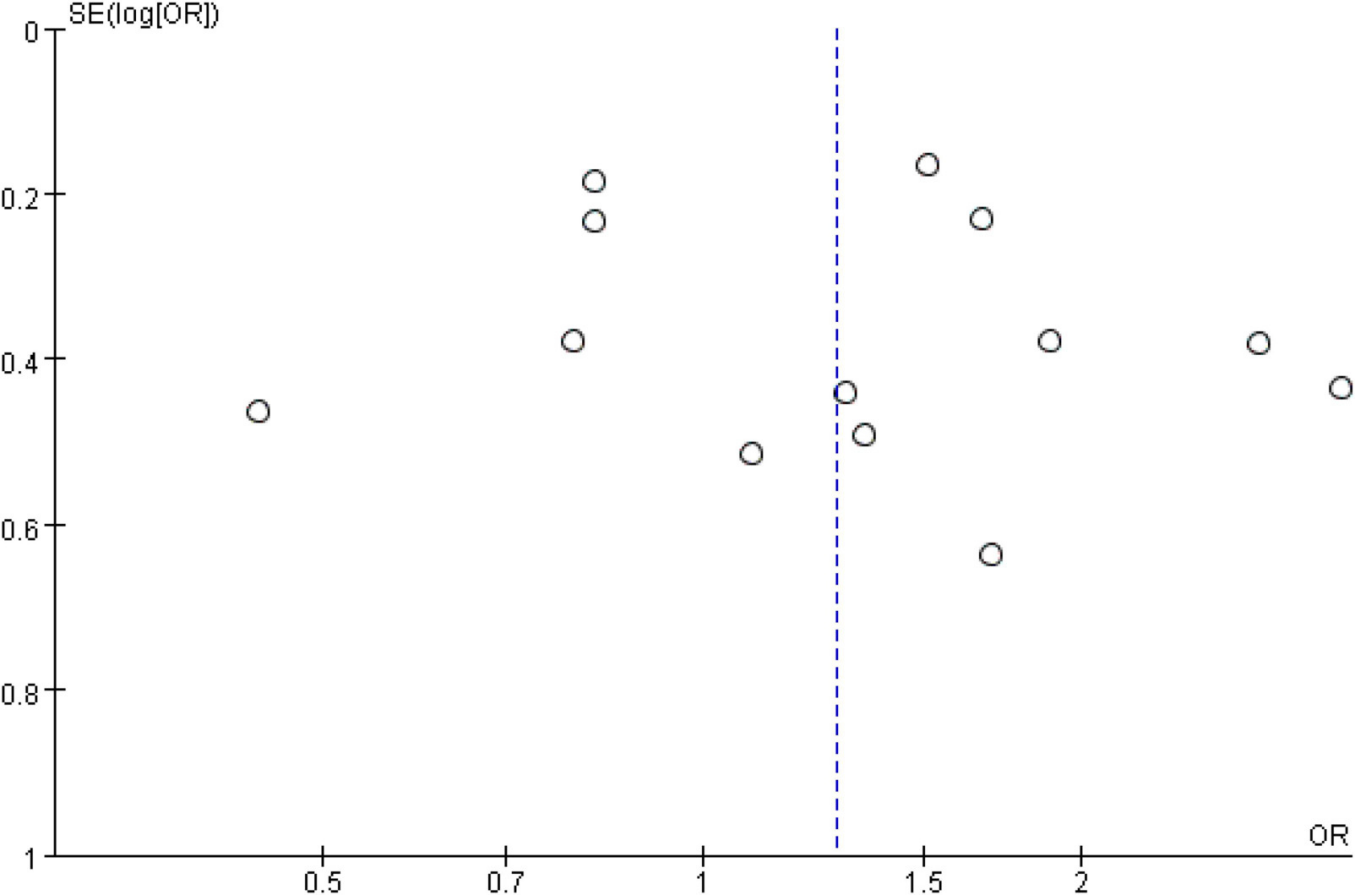
Funnel plot comparing the incidence of catheter-related thrombosis on the left and right sides of the arm port.

Funnel plots for chest ports (Figure 6) and arm ports (Figure 7) showed asymmetric distribution of included studies, suggesting potential publication bias.

## 4 Discussion

This study performed a meta-analysis of 21 relevant studies, including 10,778 patients, to comprehensively and systematically compare the incidence of CRT in left- vs. right-sided TIVAPs placed in the chest and arm. Despite including cohort studies and RCTs in our initial eligibility criteria, no such studies met all inclusion requirements (e.g., sufficient data on implantation side and CRT incidence), leading to the inclusion of only case-control studies. Overall analysis revealed no statistically significant difference in CRT incidence between left and right chest-placed TIVAPs; similarly, no such difference was observed for arm-placed TIVAPs. These findings suggest that the implantation side of TIVAPs is not a major factor associated with the development of catheter-related thrombosis in general, but may exert a subtle effect in large cohorts.

Notable variations may exist across countries in technical specifications for TIVAP implantation, perioperative nursing protocols, anticoagulant prophylaxis strategies, and patient population characteristics—all of which may modulate the risk of CRT. In subgroup analyses stratified by country, no statistically significant difference in CRT incidence was observed between left- and right-sided TIVAPs (either chest- or arm-ports) in both Chinese and non-Chinese cohorts. In contrast, subgroup analysis by sample size revealed that for studies with a sample size exceeding 500 cases, the incidence of CRT was significantly higher in left-sided vs. right-sided TIVAPs (chest- and arm-ports). None such statistically significant difference was detected in studies with a sample size of ≤500 cases. These findings imply that sample size may modify the association between TIVAP implantation side and CRT incidence. Larger sample sizes are more likely to uncover the relatively elevated CRT risk associated with left-sided TIVAP implantation, as increasing sample size mitigates the confounding effects of individual variability and other extraneous factors, thereby unmasking the inherent anatomical and hemodynamic disadvantages of left-sided implantation. Specifically, the unique angulation between the left brachiocephalic and the superior vena cava may induce reduced blood flow velocity and vortex formation in this region, which in turn promotes platelet aggregation and subsequent thrombogenesis. In smaller-sample studies, however, the inherent research uncertainty may mask this potential difference, leading to the failure to detect statistically significant variations in CRT incidence by implantation side.

A meta-analysis by Li et al. (16) found no significant difference in CRT incidence associated with peripherally inserted central catheters (PICCs) placed in different upper extremity veins. Additionally, multiple studies (17, 18, 22, 27, 35) have reported no difference in CRT risk between right- and left-sided TIVAPs, which is consistent with the overall findings of the present study. A plausible mechanistic explanation is that thrombogenesis is predominantly determined by a combination of factors—including local hemodynamic status, catheter tip position, patient coagulation profile, and postoperative activity levels—rather than by implantation side alone. Furthermore, the anatomical disparities associated with different implantation sides may be partially abrogated by the standardization of implantation techniques and advancements in catheter materials. Nevertheless, the conclusions of this study are inconsistent with those of several small-sample clinical studies, which have reported a lower CRT incidence with right-sided TIVAP implantation (14, 29). The core rationale for this finding is that the smaller angulation between the right brachiocephalic vein and the superior vena cava facilitates optimal catheter tip positioning in the vascular lumen, thereby reducing mechanical irritation to the vascular intima and lowering thrombotic risk. Wang (36) conducted a study of 700 patients with PICC placement and analyzed CRT incidence by catheter insertion site, reporting CRT rates of 11% for the right basilic vein, 21% for the right median cubital vein, 48% for the right cephalic vein, 16% for the left basilic vein, 33% for the left median cubital vein, and 59% for the left cephalic vein.

Several factors may underlie these conflicting findings. First, variations in the sample size of included studies may increase susceptibility to selection bias and random chance effects. Second, differences in the baseline characteristics of study populations—such as a higher proportion of patients with diabetes mellitus or a history of thrombosis in some cohorts—may amplify the potential impact of implantation side on thrombotic risk. Third, disparities in postoperative TIVAP maintenance protocols (e.g., standardized flushing and locking procedures, and appropriate anticoagulant use) can markedly reduce CRT incidence, which may mask the effect of implantation side.

This meta-analysis has several limitations. Despite the inclusion of 10,778 patients across the included studies, subgroup analysis by sample size confirmed that sample size modulates the study results. Future research should endeavor to further expand the total sample size, particularly by enrolling more large-sample studies (*n* > 500), to more accurately evaluate the association between TIVAP implantation side and CRT incidence. The stratification criteria for subgroup analysis were relatively simplistic, with only sample size and region used as grouping factors; this may fail to fully capture the impact of other potential confounders on CRT incidence following TIVAP implantation. Additionally, this meta-analysis exhibited inherent heterogeneity. Although subgroup analysis and sensitivity analysis were employed to explore and address heterogeneity, its residual effects could not be completely eliminated. As a literature-based meta-analysis, the present study was unable to access or uniformly adjust for key confounders of CRT reported in the original studies, including catheter material, tip position, specific anticoagulant regimens, tumor type and stage, and detailed comorbidity profiles. While some included studies accounted for these factors in their study design, adequate statistical control was not feasible at the data synthesis stage of this meta-analysis, which may have introduced bias into the estimation of the independent effect of TIVAP implantation side on CRT incidence.

## 5 Conclusions

Current evidence suggests no significant difference in CRT incidence between left- and right-sided TIVAPs. Clinicians may therefore individualize TIVAP implantation side selection based on the specific clinical characteristics of each patient. Given the limitations of the present study, future large-sample, multicenter randomized controlled trials are warranted to further elucidate the association between TIVAP implantation side and CRT incidence.

## Data Availability

The original contributions presented in the study are included in the article/Supplementary Material, further inquiries can be directed to the corresponding authors.

## Supplementary material

Supplementary Table S1 Search strategies.
