# Supplementary material for: Is there a difference in catheter-related thrombosis between left- and right-sided arm ports and chest ports?
Source: Front Surg. 2026 Feb 20;13:1735554. doi: 10.3389/fsurg.2026.1735554 (PMC12963262; doi:10.3389/fsurg.2026.1735554)
Supplement: Supplementary Table S1 — Search strategies. [file Datasheet1.docx]

| **Table S1 Search strategies** | |
| --- | --- |
| Number | Search words |
| #1 | Port[MeSH Terms] |
| #2 | TIVAP[Title/Abstract] |
| #3 | Vascular Access Port[Title/Abstract] |
| #4 | Totally implantable venous access Port[Title/Abstract] |
| #5 | implantable central venous catheter system[Title/Abstract] |
| #6 | Fully buried drug infusion device[Title/Abstract] |
| #7 | Infusion port implantation[Title/Abstract] |
| #8 | Catheter placement at the infusion port[Title/Abstract] |
| #9 | Port Catheter[Title/Abstract] |
| #10 | Catheter, Port[Title/Abstract] |
| #11 | Ports, Vascular Access[Title/Abstract] |
| #12 | Port Catheters[Title/Abstract] |
| #13 | Catheters, Port[Title/Abstract] |
| #14 | OR/1-13 |
| #15 | Venous thrombosis[MeSH Terms] |
| #16 | Thrombosis[Title/Abstract] |
| #17 | Venous Thrombosis[Title/Abstract] |
| #18 | Catheter-related blood thrombus[Title/Abstract] |
| #19 | Catheter-related thrombosis[Title/Abstract] |
| #20 | Blood thrombus[Title/Abstract] |
| #21 | Venous thrombus[Title/Abstract] |
| #22 | Venous Thromboses[Title/Abstract] |
| #23 | Thromboses[Title/Abstract] |
| #24 | Thrombus[Title/Abstract] |
| #25 | Blood Clot[Title/Abstract] |
| #26 | Blood Clots[Title/Abstract] |
| #27 | Atherothrombosis[Title/Abstract] |
| #28 | OR/15-27 |
| #29 | #14AND#28 |
